# LncRNA WWC2-AS1 functions AS a novel competing endogenous RNA in the regulation of FGF2 expression by sponging miR-16 in radiation-induced intestinal fibrosis

**DOI:** 10.1186/s12885-019-5754-6

**Published:** 2019-07-01

**Authors:** Ju-Mei Zhou, Rong Liang, Su-Yu Zhu, Hui Wang, Min Zou, Wei-Jing Zou, Shao-Lin Nie

**Affiliations:** 1 Department of Radiotherapy, Hunan Cancer Hospital & The Affiliated Cancer Hospital of Xiangya School of Medicine, Central South University, Changsha, 410013 People’s Republic of China; 2 Key Laboratory of Translational Radiation Oncology, Hunan Province Changsha, 410013 People’s Republic of China; 3 Department of Oncology, Xiangtan Central Hospital, Xiangtan, 411100 People’s Republic of China; 4 Department of Intestinal Surgery, Hunan Cancer Hospital & The Affiliated Cancer Hospital of Xiangya School of Medicine, Central South University, No.283, Tongzipo Road, Yuelu District, Changsha, 410013 Hunan Province People’s Republic of China

**Keywords:** WWC2-AS1, Radiation-induced intestinal fibrosis, ceRNA, miR-16, FGF2

## Abstract

**Background:**

Recently, long non-coding RNAs (lncRNAs) were considered as important gene expression regulators involving various biological processes. In this study, we explored the role of lncRNAs in the pathogenesis of radiation-induced intestinal fibrosis (RIF).

**Methods:**

LncRNAs were screened by microarray (Human LncRNA Array v3.0, Arraystar, Inc.) and the differentially expressed lncRNAs in RIF and non-RIF were analyzed by bioinformatics methods. The expression of WWC2-AS1/miR-16/FGF2 axis was compared on mRNA and protein level between human intestinal CCD-18Co fibroblasts cell lines and subepithelial SEMFs in response to radiation treatment. The significance of WWC2-AS1 in regulating FGF2 associated proliferation, migration, invasion and fibrosis of CCD-18Co and SEMFs by exposure to radiation was analyzed by shRNA (WWC2-AS1 shRNA) knock-down of endogenous WWC2-AS1.

**Results:**

WWC2-AS1 and FGF2 level was significantly higher while miR-16 was down-regulated in radiation-treated intestinal tissues. WWC2-AS1 more potently boosted FGF2 expression via reducing miR-16, and WWC2-AS1 shRNA remarkably inhibited FGF2 associated proliferation, migration, invasion and fibrosis of radiation treatment in vitro*,* further demonstrating physical interaction between miR-16 and WWC2-AS1 in radiation-induced fibrosis progress.

**Conclusions:**

WWC2-AS1 was highly expressed in RIF, may function as a ceRNA in the regulation of FGF2 by binding miR-16. Targeting WWC2-AS1 thus may benefit radiation-induced fibrosis treatment.

## Background

Radiation-induced intestinal fibrosis (RIF) is a common complication in patients with abdominal and pelvic malignancies who have received radiotherapy. The risk is estimated to be 5–20% within 6 months to 6 years [1, 2]. Patients suffer from abdominal pain, changing bowel habits with constipation and many other symptoms owing to fibrosis and stricture formation [3]. Excessive fibrosis may lead to ano-rectal compliance lost and increased obstruction risk. The mechanisms underlying radiation-induced fibrosis remain poorly understood, and currently no effective therapies or drugs are available.

The multifunctional cytokine fibroblast growth factor 2 (FGF2) orchestrates an intricate signaling network to modulate radiation-induced fibrosis. FGF2 exacts its role in radiation-induced fibrosis through enhancing interstitial cell proliferation and stimulating trans-differentiation of fibroblasts into myofibroblasts [4, 5] partially through its ability to induce the epithelial-mesenchymal transition (EMT) [6, 7]. The EMT has been shown to be of critical importance in damage repair and organ fibrosis [8–10]. In addition to interstitial cell proliferation, these cells (especially myofibroblasts) produce excess collagen and other extracellular matrix (ECM) components, which accumulate in the rectal wall over a long period of time. Thus, elucidating the special upstream regulatory mechanism of FGF2 will aid in the search for specific inhibitors of the different FGF2-dependent pathways for RIF.

According to the latest reports, long non-coding RNAs (lncRNAs) may be involved in a variety of diseases, including tumors [11], neurodegenerative diseases [12], cardiovascular diseases [13], and fibrosis [14, 15]. Moreover, lncRNAs may function as competing endogenous RNAs (ceRNA) by binding to specific microRNAs (miRNAs) to modulate their target genes [16]. Accumulating evidence has indicated that miRNAs regulate expression of TGF-β, CTGF [17] and FGF2 [18], which play vital roles in fibrosis signaling pathway. Moreover, recent studies confirmed that miRNAs were involved in radiation-induced fibrosis [19, 20]. However, more studies are needed for a better understanding of the complicated relationship between miRNAs and RIF.

The present work provides evidence for a ceRNA network among WWC2-AS1/FGF2 and miR-16, which sheds new light on the treatment of RIF. In this study, we first identify WWC2-AS1 as FGF2 related lncRNA with higher expression in RIF than in paired adjacent tissues. Then, we found that no isoforms of WWC2-AS1 were identified after bioinformatics analysis, although we did find an external reference matched to transcript ENST00000511846, namely, ENSG00000251128, which locates on the same region with ENST00000511846 on Chromosome 4: 183,233,628-183,240,634 reverse strand. Down-regulating of WWC2-AS1 significantly inhibited proliferation, migration, invasion and fibrosis induced by radiation treatment in vitro. Our data indicate that WWC2-AS1 in intestine could directly interact with miR-16-5p and function as a sponge for miR-16-5p to modulate FGF2 expression. Thus, WWC2-AS1 functions as a ceRNA in the regulation of the expression of FGF2 through competition for miR-16 in RIF.

## Methods

### Tissue sampling, cell culture and shRNA construction

All experimental protocols involving human tissue samples were reviewed and approved by the Ethics Committee of The Affiliated Cancer Hospital of Xiangya School of Medicine, Central South University. A total of 130 colorectal cancer patients were recruited into this study. Of these patients, 75 had RIF and 55 were non-RIF controls, their mean ages were 52.7 ± 9.3 yr. and 59.2 ± 3.5 yr., respectively. All participants were diagnosed with locally advanced colorectal cancer, and a history of chronic proctitis and abdominal/pelvic radiation therapy was excluded. The patients in the RIF group were given neo-adjuvant chemoradiotherapy (50 Gy in 25 to 28 fractions to the tumor or lymph node volumes and oral capecitabine at 1650 mg/m2/5 days per week during radiotherapy) and received a total mesorectal excision (TME) operation 9 to 12 weeks after the end of the neo-adjuvant chemoradiotherapy. The patients of the control group only received the TME operation.

Human intestinal CCD-18Co fibroblasts cell lines were obtained from the ATCC and intestinal subepithelial (SEMFs; passages 3–8) isolated from colonic tissues. Both CCD-18Co and SEMFs were ultimately incubated in Dulbecco’s modified Eagle medium (DMEM, high glucose) (Gibco, Grand Island, NY, USA) containing 10% fetal bovine serum (FBS; Gibco) and 100 mg/ml penicillin/streptomycin (P/S) (Invitrogen, Carlsbad, CA, USA) and cultured in a 5% (v/v) CO_2_ humidified atmosphere at 37 °C. Non-adherent and adherent cells appeared in the culture flasks were removed every 72 h until myofibroblasts foci appeared. Intestinal myofibroblasts were cultured in DMEM+ 10% FBS + P/S. Ionizing radiation (IR) was done with 6 MV photons, 8 Gy/min by a linear accelerator (Varian, America) at room temperature.

Short-hairpin RNA (shRNA) against human WWC2-AS1(WWC2-AS1 shRNA) was constructed into pAdTrack-CMV system (Addgene, Cambridge, MA, USA) following manufacturer’s instructions. Adenovirus was generated and propagated in HEK293 cells. The titer of the adenovirus was 1.0 × 10^10^ plaque-forming units (pfu)/ml.

### Histological examination

Upon isolation, the RIF and non-RIF tissues were fixed in 4% paraformaldehyde at 4 °C overnight. Paraffin embedded sections (4-μm) were then prepared. Hematoxylin and eosin (HE) staining were conveyed by HE satining kit (Sigma Aldrich, St. Louis, MO, USA) according to the manufacturer’s instructions.

### Microarray and bioinformatics analysis

LncRNA microarrays of RIF or non-RIF tissues were conveyed by KangChen Biotech (Shanghai, China). Hybridization was conveyed using Human LncRNA Array v3.0 (Arraystar, Rockville, MD, USA). Array images were analyzed using Agilent Feature Extraction software. We performed quantile normalization and data processing using GeneSpring GX v11.5.1 (Agilent Technologies, Santa Clara, CA, USA). Differential expression of lncRNA was analyzed by volcano plot.

### RNA-seq

Three RIF and three non-RIF samples were analyzed by RNA-seq. Total RNA was extracted with TRIzol. Briefly, mRNA was isolated from the total RNA, amplified and transcribed into fluorescent cRNA. The fluorescent labeled cRNAs were then purified with the RNeasy Mini Kit (Qiagen). Fragmentation of labeled cRNA was down by mixing 1μg cRNA with 5ul 10 × blocking agent and 1ul 25× fragmentation buffer. Samples were then mixed with25ul 2× GE Hybridization buffer after incubation at 60 °C for 30 min. 50 μl of hybridization solution was loaded on microarray slide and incubated for 17 h at 65 °C, washed, fixed and scanned using the Agilent DNA Microarray Scanner (part number G2505C).

### Real-time quantitative PCR (RT-qPCR)

Total RNA was extracted by Trizol, cDNA was then synthesized using High-Capacity cDNA Reverse Transcription Kit (Applied Biosystems, CA, USA). RT-PCR was conveyed with ABI7500 platform (Applied Biosystems). Primers used in this research were as follows: human WWC2-AS1 F:5′-CAACTGTCAACTTGCTCCTTCTGG3’, R: 5′- CTGGGATTCTGCTTTCCTCTGTG’; human FGF2 F:5’CCAATACTCGTTTTGCCTCTA3’, R:5’CATGTTTCTGTGACTTCCTCTTC3’; human β-actin F:5′-CACCAACTGGGACGACAT-3′, R:5′-ACAGCCTGGATAGCAACG-3′. Each reaction was set up in triplicate and the relative expression level of WWC2-AS1 and FGF2 were calculated using 2^-ΔΔCt^ method [14].

### Western blot

CCD-18Co or SEMFs cells were lysed with RIPA buffer (Thermo Fisher Scientific, MA, USA). Protein concentration were detected by BCA kit (Thermo Fisher Scientific, NY, USA). Samples were separated on SDS-PAGE gel and transferred to polyvinylidene difluoride (PVDF) membrane. Membrane was blocked and incubated with primary antibodies at 4 °C overnight. Antibodies used in this research were as follows (all from Abcam, MA, USA): FGF (ab16828), α-smooth muscle actin (α-SMA; ab5694), Collagen I (ColI; ab34710), GAPDH (ab37168). Membranes were washed 3 times by TBST and incubated with 2nd antibodies at room temperature for 2 h, and washed for another 3 times by TBST. Signal was detected by ECL method (Thermo Fisher Scientific) following manufacturer’s instructions.

### MTT assay

CCD-18Co or SEMFs cells were transfected with NC or sh-WWC2-AS1. 5 × 10^5^ cells per well were seeded into 96-well plates (Corning, Corning, NY, USA). 20 μl of MTT agent (5 mg/ml) was added into each well and incubated at 37 °C for 4 h. Supernatants were discarded and replaced with 150 μl DMSO. Absorbance was measured at 490 nm and reference was detected under 630 nm.

### Flow cytometry analysis

Briefly, 1 × 10^7^ per ml NC- or WWC2-AS1 shRNA-transfected CCD-18Co or SEMFs cells was incubated for 30 min on ice with PI and Annexin-V after washing. The fluorescence intensity from the samples was determined using a FACS Calibur flow cytometer (Becton Dickinson, San Jose, CA, USA) and calculated using FlowJo.

### Transwell assay

The successfully NC- or WWC2-AS1 shRNA-transfected CCD-18Co or SEMFs cells were seeded into the upper chambers (10 × 10^5^ in 200 μl serum-free medium) of cell culture inserts (24-well type, 8 μm pore size, Millipore, MA). 24 h later, the upper chambers were removed. Cells in the lower chamber were fixed with 4% paraformaldehyde and stained with 0.25% crystal violet (Beyotime, China). The number of cells in 5 random fields was counted under a microscope.

### RNA immunoprecipitation

MiR-16 associated with WWC-AS1 was purified with Maltose-binding protein (MBP)-affinity assay. Three MS2 binding sites were connected to the 3′ end of WWC-AS1. To obtain miR-16 associated with the MS2-tagged WWC-AS1, the CCD-18Co or SEMFs were transfected with WWC-AS1-3MS2 for 48 h and 10^7^ × cells were subjected to each RIP assay.

### Dual luciferase reporter assay

WWC-AS1 were cloned into pmirGLO vector. The mutant WWC-AS1 was synthesized using QuickChange Lightning Multi Site-Directed Mutagenesis Kit (Agilent Technologies, Palo Alto, CA, USA). The wild type (Wt-WWC-AS1) or mutant (Mut-WWC-AS1) was co-transfected with miR-16 mimic or control into CCD-18Co or SEMFs cells using Lipofectamine 3000 transfection reagent (Thermo Fisher Scientific). 48 h later, luciferase intensity were detected and analyzed by Dual Luciferase Reportor Assay System (Promega, Madison WI, USA) following manufacturer’s instructions. All experiments were performed in triplicate.

### Statistical analysis

All data were presented as mean ± SD and repeated at least three times experiments, at last analyzed by SPSS 22.0 software. Variance between two groups or among three group were analyzed by student’s *t* test and a one-way ANOVA, respectively. *P <* 0.05 was considered statistically significant.

## Results

### lncRNA WWC2-AS1 is frequently over-expressed in RIF

**Fig. 1:**
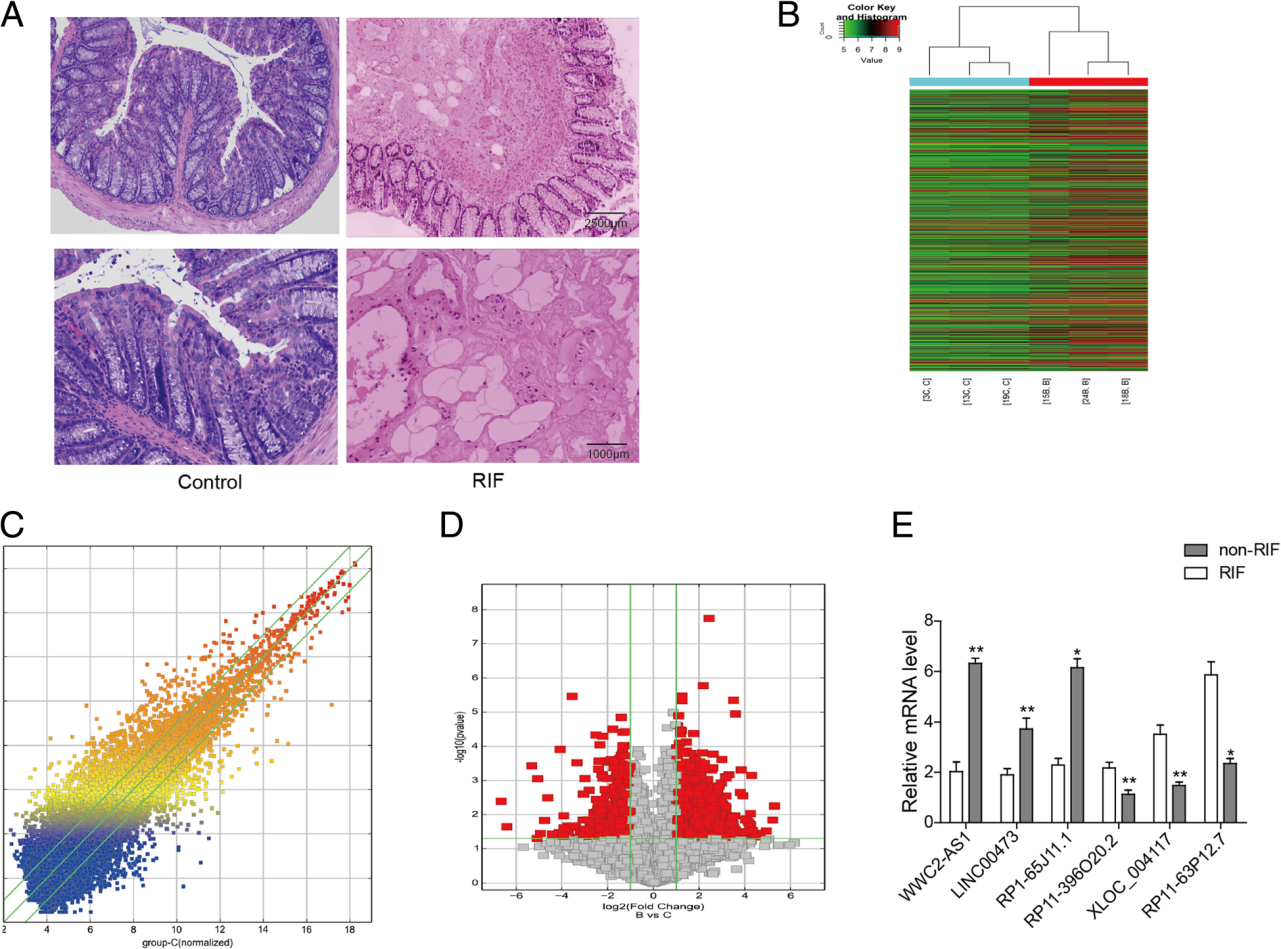
Microarray screening of alterations in the lncRNA expression profiles. **a** RIF and non-RIF tissues stained with H&E (× 100) (× 200). **b** The clustering analysis shows the relative expression levels of the lncRNAs. Red represents up-regulated lncRNAs, and green represents down-regulated lncRNAs. **c** Scatter-plots show the differential expression profiles of the lncRNAs in the RIF and non-RIF tissues. Values plotted on the Y axis are the standard expression levels of the lncRNAs in the RIF tissue samples, and the values plotted on the X axis are the standard expression levels of the lncRNAs in the non-RIF tissue samples. The values above the top green line and below the bottom green line indicate lncRNAs with 2.0-fold differential expression. **d**Volcano plots show differential expression pattern of RIF and non-RIF groups. **e** RT-qPCR to confirm the differentially expressed lncRNAs between the RIF and non-RIF tissue samples. *P < 0.05, **P < 0.01, compared to non-RIF tissues

To comprehensively analyze the changes of lncRNAs in RIF, we analyzed tissues from three individuals with RIF and paired non-RIF controls by H&E, as seen in Fig. 1a, the submucosa is thickened, fibrous hyperplasia and obvious lesions are around the adipocytes and vessels with glassy and collagenous changes. Hierarchical clustering identified 1622 lncRNAs (1038 up-regulated and 584 down-regulated lncRNAs) with differential expression between the two groups of samples subjected to lncRNA microarray analysis. Expression threshold was set at greater than two-fold with *P* < 0.05. In total, the expression of 76 lncRNAs (54 up-regulated and 22 down-regulated) exhibited greater than ten-fold differences between the two groups of samples (*P* < 0.05) (Fig. 1b-d). To validate microarray results, we used RT-qPCR to analyze the expression of 6 lncRNAs in 15 RIF and corresponding adjacent non-RIF tissue samples. LncRNA WWC2-AS1 was most significantly differently expressed in RIF against non-RIF tissues (Table 1, Fig. 1e). Thus, lncRNA WWC2-AS1 was selected for further study for its role in regulating RIF progression.

**Table 1:** Relative expression of lncRNAs in rectal tissue of two groups (Only *P*<0.05 were showed)

lncRNAs	2 ^- △△ CT^ mean			*P* value
	RIF	non-RIF	Ratio	
WWC2-AS1	6.322 ± 0.21	2.039 ± 0.38	3.10	0.002
LINC00473	3.727 ± 0.43	1.903 ± 0.25	1.96	0.003
RP1-65 J11.1	6.157 ± 0.35	2.289 ± 0.27	2.67	0.013
RP11-396O20.2	1.127 ± 0.17	2.176 ± 0.23	1.93	0.001
XLOC-004117	1.479 ± 0.14	3.514 ± 0.37	2.38	0.005
RP11-63P12.7	2.357 ± 0.19	5.872 ± 0.51	2.49	0.027

### WWC2-AS1 is closely related to FGF2 signaling in RIF

**Fig. 2:**
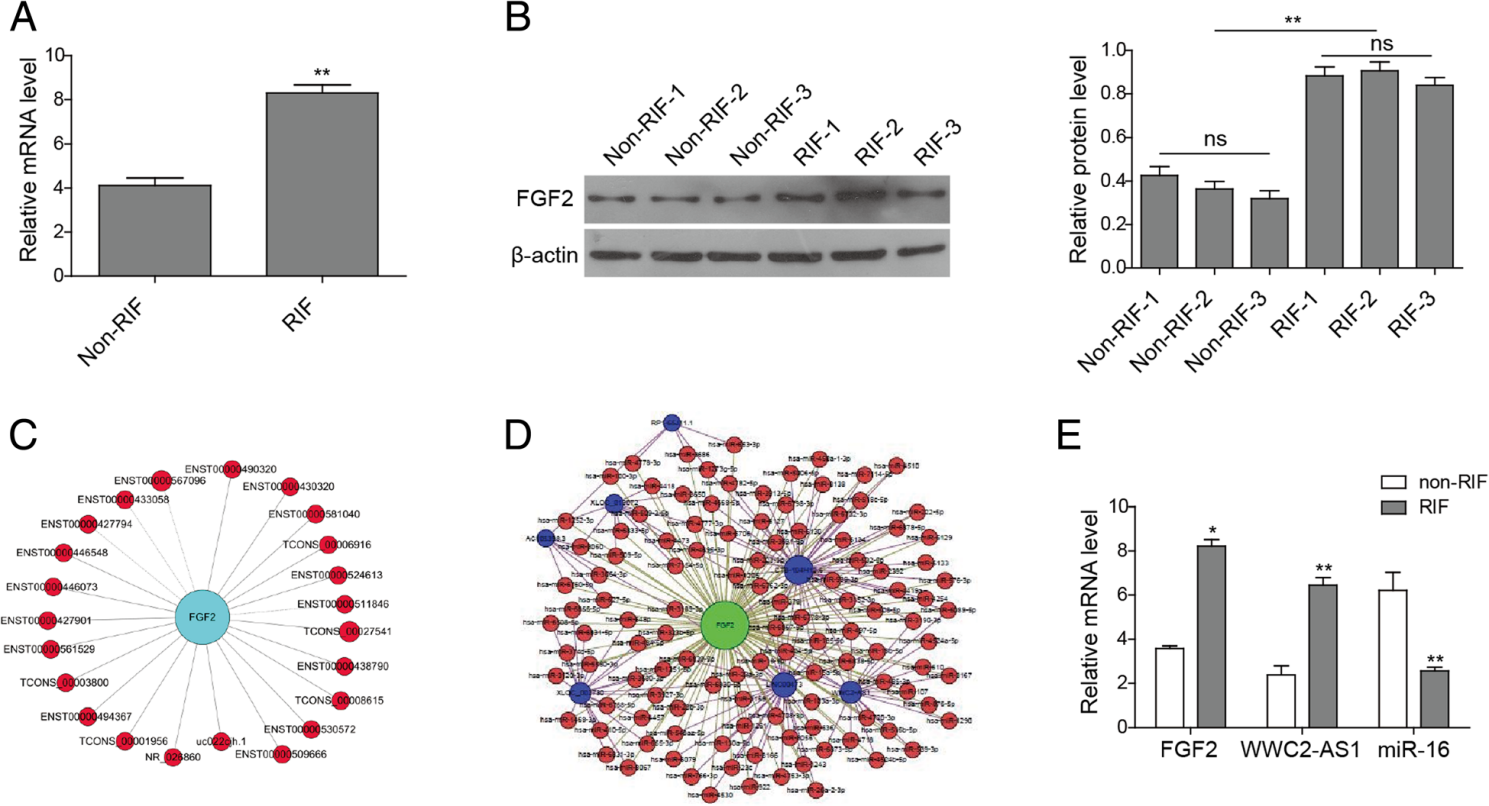
FGF2 expression pattern and CNC network in RIF and non-RIF tissues. **a** RT-qPCR analysis of FGF2 mRNA expression between the RIF and non-RIF tissue samples. **b** Western blotting analysis of FGF2 expression. **c** FGF2-associated lncRNA network was performed by GO analysis. The blue node represents the FGF2 mRNA, the red nodes represent lncRNA related with FGF2. ENST00000511846 represents lncRNA-WWC2-AS1. Lines represent positive correlation and dotted lines represent negative correlation. **d**The TargetScan, miRanda, and miRBase databases were used to predict the interactions of FGF2, miRNAs, and lncRNAs and build the lncRNA-miRNA-FGF2 network diagram. The green node represents the FGF2 mRNA, the blue nodes represent different lncRNAs, and the red nodes represent different miRNAs. The lines between the lncRNAs, miRNAs, and FGF2 represent direct interactions between lncRNAs and miRNAs, with FGF2 as the target of the miRNAs. **e** RT-qPCR analysis of FGF2 mRNA, WWC2-AS1 and miR-16 expression in the RIF and non-RIF tissues samples. The data are expressed as the mean ± SEM, *P < 0.05, **P < 0.01, compared to non-RIF tissues

By GO analysis, we found multiple genes near lncRNAs that might encode key factors of the fibrosis signaling pathway, such as FGF2. To confirm whether FGF2 screened from lncRNA chips is related to WWC2-AS1 in RIF, we assess the FGF2 level using RT-qPCR and Western blot, and found expression of FGF2 in RIF was significantly higher than control tissues (Figs. 2a and 2b), indicating FGF2 in RIF group was up-regulated. To systematically select lncRNAs that might regulate FGF2 mRNA expression, we performed a coding-non-coding gene co-expression (CNC) analysis, which showed multiple lncRNAs, including WWC2-AS1, LINC00473, and XLOC_002730, were closely related to the FGF2 signaling pathway and the correlation coefficient between lncRNA WWC2-AS1 and FGF2 was 0.95(Fig. 2c). In addition, we used the TargetScan, miRanda and miRBase databases to construct a ceRNA network for RIF. The results showed lncRNAs WWC2-AS1, LINC00473, XLOC_002730 directly interacted with multiple miRNAs, and the correlation coefficients for miR-16 with WWC2-AS1 and FGF2 were both 0.95(Fig. 2d). Thus, we chose miR-16 as our potential candidate miRNA and constructed the WWC2-AS1/miR-16/FGF2 ceRNA network.

Finally, we examined the WWC2-AS1, miR-16 and FGF2 expression levels by RT-qPCR in 60 RIF and 40 non-RIF tissue specimens. Our data showed that WWC2-AS1 and FGF2 were over-expressed, while miR-16 was down-expressed in the RIF tissues compared with the non-RIF tissues (Table 2, Fig. 2e) (*P* < 0.05), which was consistent with previous research results [21, 22]. Therefore, the WWC2-AS1//miR-16/FGF2 expression trends were consistent with the definition of a ceRNA.

**Table 2:** Relative expression of FGF2, WWC2-AS1 and miR-16 in rectal tissue of two groups

Objective	2 ^- △△ CT^ mean			P value
	RIF	non-RIRF	Ratio	
FGF2	8.231 ± 0.29	3.579 ± 0.12	2.30	0.016
WWC2-AS1	6.453 ± 0.34	2.381 ± 0.41	2.71	0.008
miR-16	2.560 ± 0.17	6.221 ± 0.82	2.43	0.003

### Down-regulation of WWC2-AS1 inhibited colorectal cells proliferation and invasion, migration and fibrosis in response to radiation

**Fig. 3:**
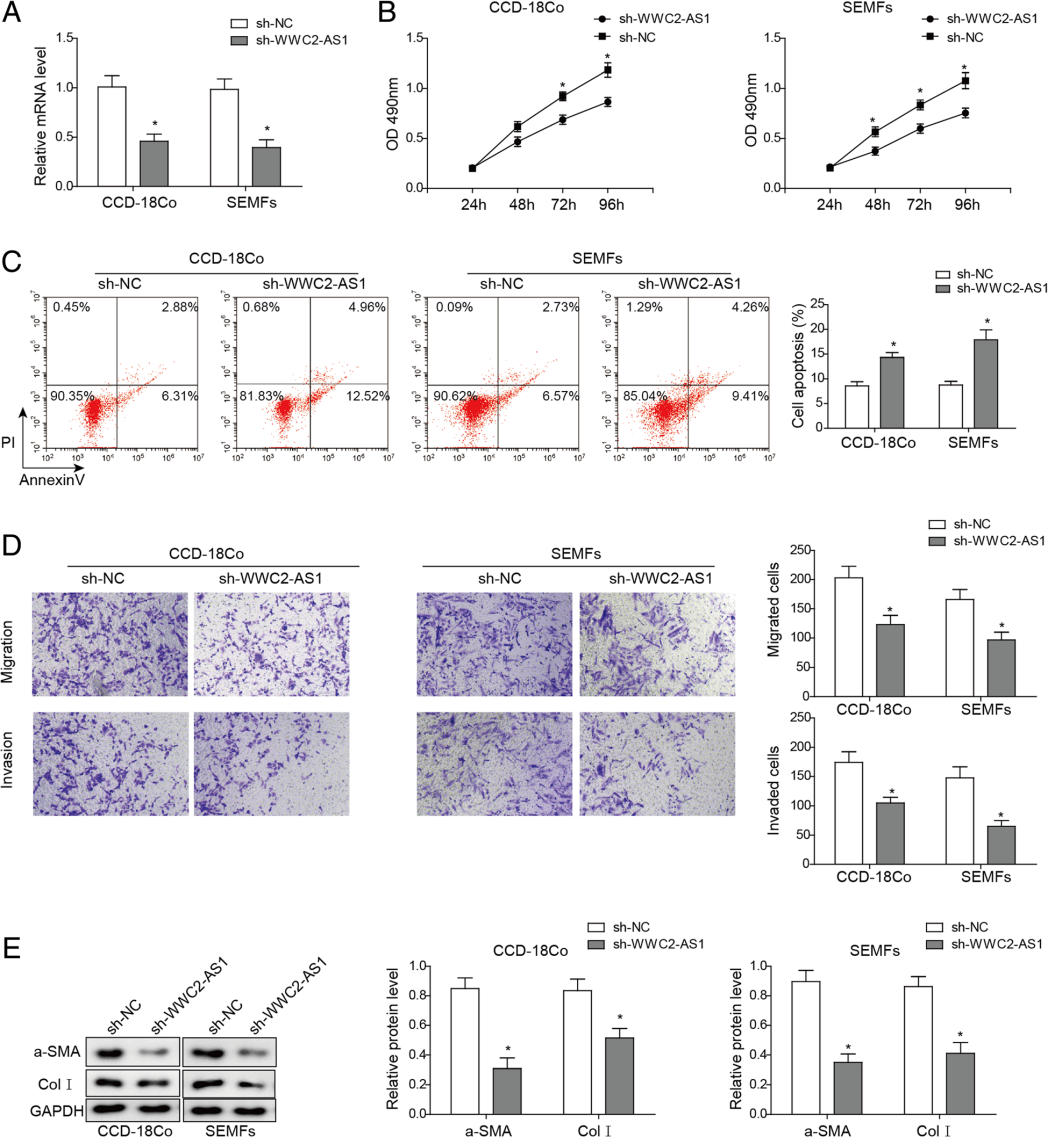
Knocking down WWC2-AS1 promotes proliferation, migration, invasion and fibrosis of colorectal cells in response to radiation. **a** Relative expression of WWC2-AS1 in CCD-18Co and NCM-460 cells with or without sh-WWC2-AS1 as indicated. **b** Cell viability was detected by MTT assay after WWC2-AS1 was knocked down in CCD-18Co and SEMFs cells. **c** Cell apoptosis was detected by FACS with AnnexinV. **d** Invasion and migration of cells were detected by Transwell assay and statistical results were shown in columns. **e** α-SMA and ColI in Cells treated as described in A were detected by Western Blot. Relative intensity was normalized to GAPDH and shown in columns. *P < 0.05, compared to control group

To validate our findings, we constructed shRNA against WWC2-AS1, and transfected intestinal CCD-18Co and SEMFs cell lines with sh-WWC2-AS1 or shNC. The cells were all exposed to radiation with 8 Gy for 12 h. WWC2-AS1 mRNA level was detected by RT-qPCR, and expression of WWC2-AS1 could be effectively reduced by shRNA (Fig. 3a). Importantly, down-regulation of WWC2-AS1 significantly inhibited cell proliferation (Fig. 3b) and induced cell apoptosis (Fig. 3c). In addition, migration and invasion were inhibited by sh-WWC2-AS1 via Transwell assay (Fig. 3d), which agreed with previous results. Finally, radiation induced fibrosis could also be alleviated by sh-WWC2-AS1, given decreased fibrosis related protein α-SMA and ColI in sh-WWC2-AS1 groups in both CCD-18Co and SEMFs cell lines (Fig. 3e).

### WWC2-AS1 regulates FGF2 via competing for miR-16 AS ceRNA

**Fig. 4:**
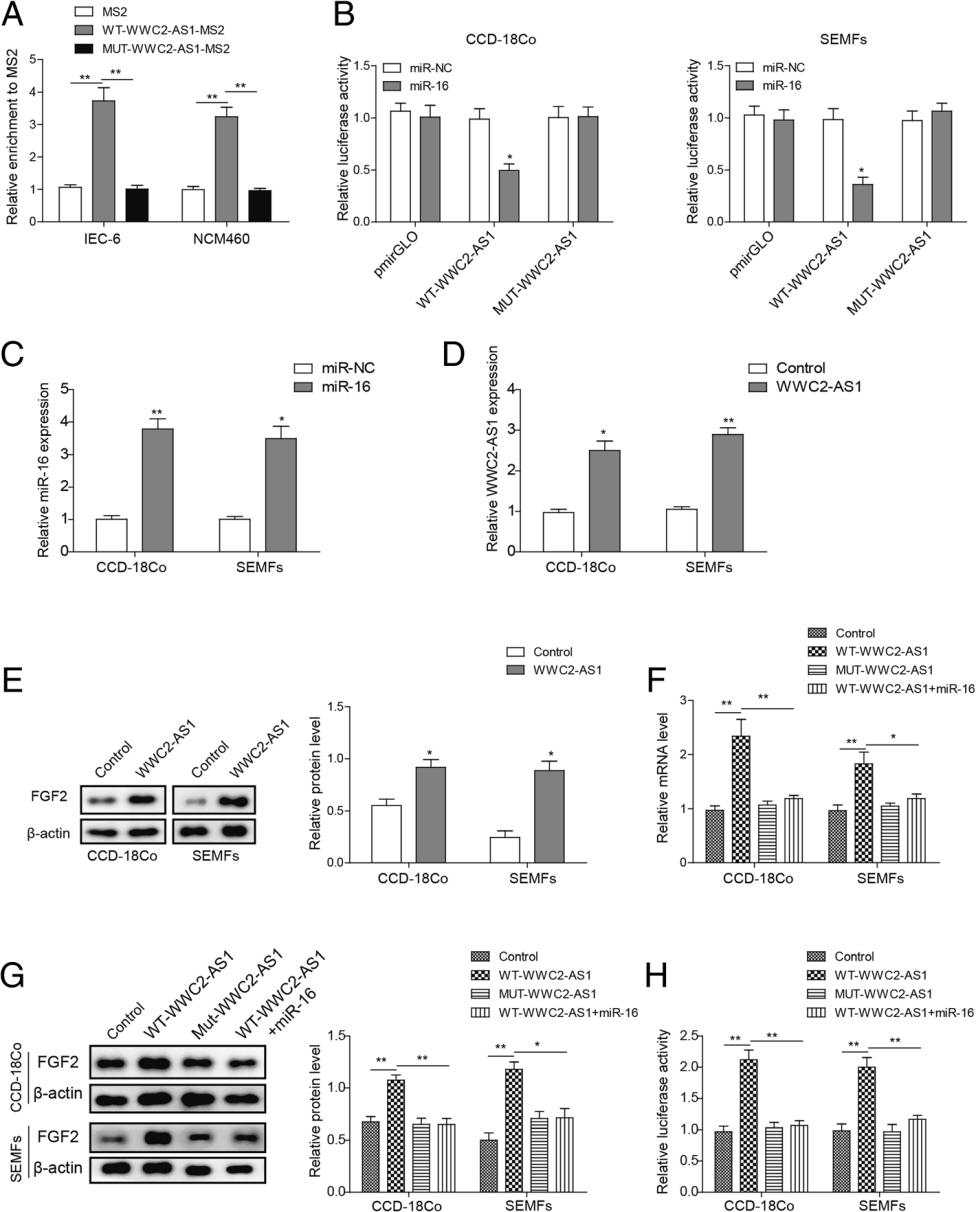
lncRNA WWC2-AS1 is physically associated with miR-16 and functions as a ceRNA of FGF2. **a** MS2-RIP followed by RT-qPCR to detect endogenous miR-16 associated with WWC2-AS1. **b** Luciferase activity in CCD-18Co and SEMFs cells co-transfected with miR-16 and luciferase reporters containing nothing (pmirGLO), Wt-WWC2-AS1 or Mut-WWC2-AS1. **c** The mRNA levels of miR-16 were determined by RT-qPCR in CCD-18Co and SEMFs cells transfected with miR-16. **d** The mRNA levels of WWC2-AS1 in control (Con) and WWC2-AS1 overexpression (WWC2-AS1) cells were determined by RT-qPCR. **e** The protein levels of FGF2 in CCD-18Co and SEMFs transfected with control and WWC2-AS1 were determined by Western Blot. Statistical analysis was displayed in columns. **f** The relative mRNA levels of FGF2 in Wt or Mut WWC2-AS1 overexpressed cells with or without overexpression of miR-16. **g** The relative protein levels of FGF2 in Wt or Mut WWC2-AS1 overexpressed cells with or without miR-16. Statistical analysis was displayed in columns. **h** The relative luciferase activity of FGF2 3’UTR in Wt or Mut WWC2-AS1 overexpressed cells with or without of miR-16. *P < 0.05, **P < 0.01, compared to control group

To investigate whether the lncRNA WWC2-AS1 could directly affect miR-16, RIP assay was performed to convey in CCD-18Co and SEMFs cells. The heavy enrichment of wt-WWC2-AS1-MS2 strongly indicates that miR-16 could directly interact with WWC2-AS1, and such interaction was abrogated when mutation into WWC2-AS1 was introduced (Fig. 4a), indicating a sequence dependent interaction between miR-16 and WWC2-AS1. Luciferase reporter assay further validated the finding in both CCD-18Co and SEMFs cells (Fig. 4b). These findings supported our hypothesis that WWC2-AS1 could physically interact with miR-16.

However, whether this interaction contributed to the regulation of FGF2 remains enigma. We therefore over-expressed miR-16 or WWC2-AS1 in CCD-18Co and SEMFs cell lines (Fig. 4c-d), and detected the relative expression of FGF2 in both cell lines, which was significantly elevated by WWC2-AS1, validating a regulatory effect between WWC2-AS1 and FGF2 (Fig. 4e). We next tried to reveal whether WWC2-AS1 regulated FGF2 through miR-16. Relative expression of FGF2 was enhanced by wt-WWC2-AS1 instead of mut-WWC2-AS1, what’s more, when we introduced ectopic miR-16 together with WWC2-AS1, expression of FGF2 was much decreased compared with WWC2-AS1 transfected only group (Fig. 4f-g). We also conveyed FGF2 luciferase reporter assay, which also exerted similar results (Fig. 4h). All these results lead to one conclusion that WWC2-AS1 functions as a ceRNA of FGF2, regulating its expression by sponging miR-16.

### WWC2-AS1 promotes fibrosis via FGF2 in response to radiation

**Fig. 5:**
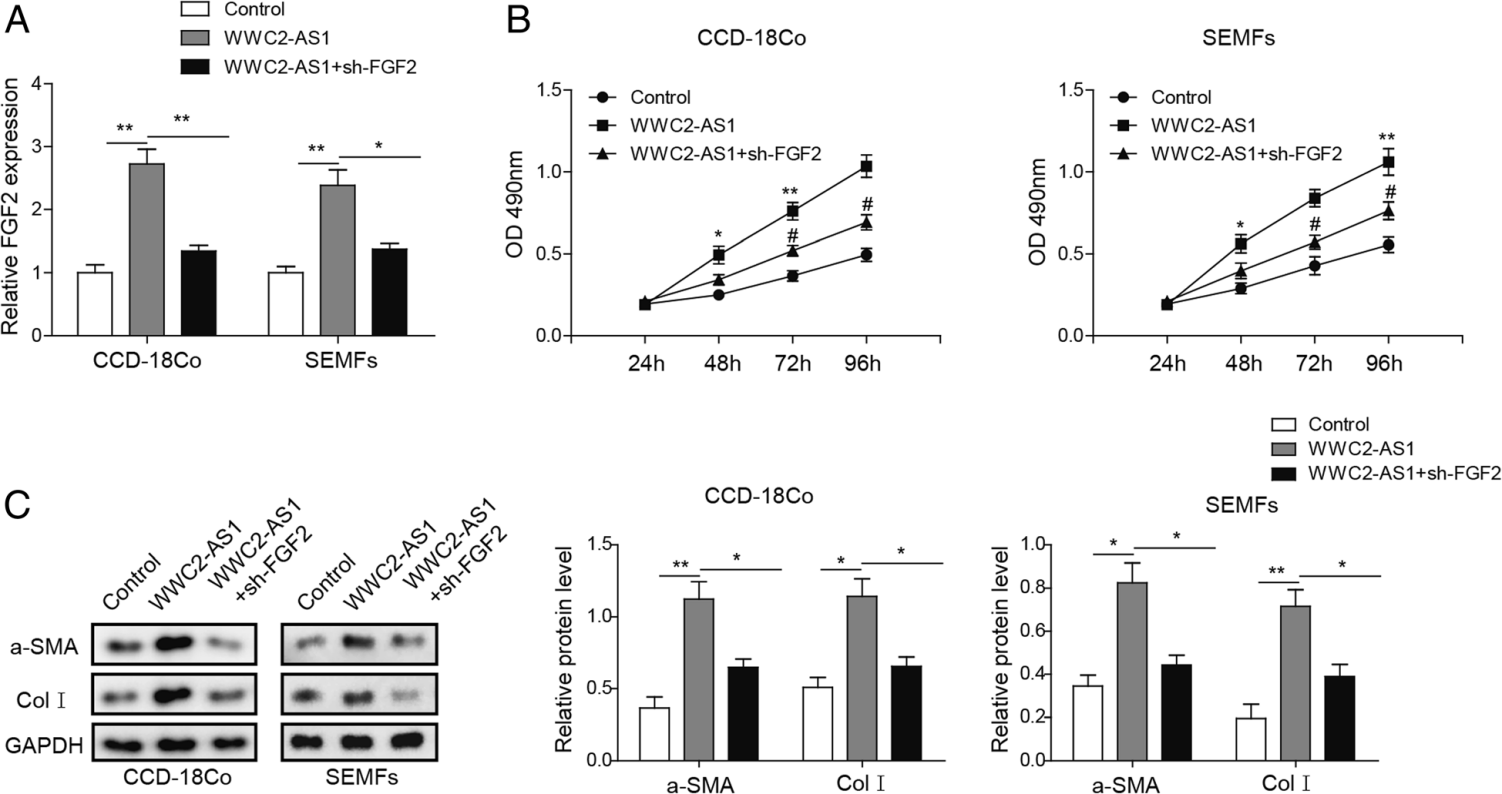
WWC2-AS1 modulates proliferation and fibrosis via FGF2. **a** The relative expression of FGF2 in WWC2-AS1 overexpression cells with or without shRNA against FGF (sh-FGF2). **b** Cell viability of CCD-18Co and SEMFs cells with WWC2-AS1 overexpression cells with or sh-FGF2 were detected by MTT. **c** SMA and ColI expression were detected by Western blot and statistical analysis was displayed in columns. *P < 0.05, **P < 0.01, compared to control group

We next tried to reveal whether WWC2-AS1 is responsible for RIF via FGF2. CCD-18Co and SEMFs was transfected with WWC2-AS1 with or without FGF2-shRNA by exposure to radiation with 8 Gy, and WW2-AS1 could dramatically enhance FGF2 expression (Fig. 5a). Viability of these cells was also detected and results indicated that decreased FGF2 inhibited proliferation (Fig. 5b). We also analyzed fibrosis by detecting fibrotic marker α-SMA and ColI, the promotion of fibrosis was remarkably abolished with decreased FGF2 expression (Fig. 5c). All these findings indicate that WWC2-AS1 could promote fibrosis via FGF2 in response to radiation*.*

## Discussion

At present, the specific mechanism of RIF is mostly unknown, and surgical resection is the only effective treatment for fibrosis. Thus, better understanding of the mechanisms of RIF is important for developing treatment strategies. In our study, we showed that lncRNA-WWC2-AS1 functions as a ceRNA to regulate FGF2 by sponging miR-16. This process is critical in promoting RIF development by regulating proliferation, apoptosis, invasion and migration.

The lncRNAs used to be considered as transcriptional “noise”. With deepening research, evidence had indicated that lncRNAs were important regulator of various biological processes, such as dosage compensation effects, cell cycle regulation, epigenetic regulation and cell differentiation. However, previous studies [23–27] have demonstrated that lncRNAs are dysregulated in fibrotic tissues, which suggests that lncRNAs are important regulators of the development of tissue fibrosis. For example, Cao et al. [28] found that up to 568 lncRNAs were differentially expressed in bleomycin-induced lung fibrosis compared with the normal lung. Indeed, using a third generation lncRNA microarray, we identified 1038 up-regulated and 584 down-regulated lncRNAs in RIF compared with normal tissues. Differences of the lncRNA transcriptomes between normal and fibrotic tissues suggest that lncRNAs may be valuable biomarkers and regulators for RIF initiation and progression.

After confirming that FGF2/FGFR was an important signaling pathway in RIF, we speculated that some of the lncRNAs might regulate the process of colorectal fibrosis by modulating FGF2. Using CNC analysis, we found a number of lncRNAs that might regulate FGF2. Combined with the qRT-PCR results, we identified lncRNA WWC2-AS1 as our potential candidate for further study.

The mechanism by which lncRNAs impact the expression of their target genes is unclear. Recently, a group of American researchers proposed that lncRNAs might function as ceRNAs to sponge miRNAs, protecting miRNA targets [29, 30]. Based on the ceRNA theory and increasing evidence, we hypothesized that WWC2-AS1 could also serve as a ceRNA in the regulation of FGF2 in RIF. Therefore, we aimed to reveal its interactions with miRNA.

Though the TargetScan, miRanda and miRBase databases, we constructed a ceRNA network (lncRNA-mRNA-miRNA) for RIF. We found that miR-16 could combine with WWC2-AS1. MiR-16 was first reported to be decreased in chronic lymphocytic leukemia (CLL) due to the deletion in chromosome 13q14 and inhibited apoptosis through targeting Bcl-2 [31]. A study showed [32] that miR-16 inhibited inflammation by regulating programmed cell death 4 (PCD4) to adjust macrophage activation. Additionally, miR-16 over-expression significantly reduced FGF2 mRNA expression and inhibited nasopharyngeal carcinoma cell proliferation and migration [21]. This study demonstrated that FGF2 was a target gene of miR-16, and agreed with findings in colorectal carcinoma [22]. Consulting the literature revealed that miR-16 regulated FGF2 expression. Previous studies support our theoretical hypothesis. In our study, we verified obvious over-expression of WWC2-AS1 and FGF2 and under-expression of miR-16 in RIF tissues compared with non-RIF tissues. The diverse expression patterns of WWC2-AS1, FGF2 and miR-16 in RIF tissues in our study suggest that WWC2-AS1 may act as a ceRNA by sponging miR-16 to promote the development of RIF.

## Conclusions

To confirm our hypothesis, we knocked down WWC2-AS1 in colorectal cells. Direct interaction between WWC2-AS1 and miR-16 was confirmed by RIP and dual luciferase reporter assays, this finding strongly supported our ceRNA theory that WWC2-AS1 could compete for miR-16. In other results, WWC2-AS1 over-expression decreased miR-16 level, resulting in up-regulated expression of FGF2. Till now, all ceRNA network was confirmed. We then tried to evaluate whether this ceRNA network could affect RIF progress. Bioinformatics analysis results were then validated by molecular cell biology experiments. Down-regulation of WWC2-AS1 inhibited colorectal cell proliferation, cell migration and invasion, together with increased apoptosis and decreased fibrosis. All these were considered hallmarks of RIF, thus, our results indicated that WWC2-AS1 could promote RIF.

The regulatory effect of WWC2-AS1 on miR-16 and FGF2 were also validated respectively by RIP and RT-qPCR assays. In intestine cells treated with radiation, WWC2-AS1 over-expression indeed sponged miR-16 and enhanced FGF2, leading to promote fibrosis.

## Data Availability

All data generated or analyzed during this study are included in this published article and all raw data in this study is available upon request, you should contact the first author by e-mail..

## Abbreviations

CLL: chronic lymphocytic leukemia
CNC: coding-non-coding gene co-expression
ECM: extracellular matrix
EMT: epithelial-mesenchymal transition
FGF2: fibroblast growth factor 2
GO: gene ontology
HE: hematoxylin and eosin
IR: Ionizing radiation
MBP: Maltose-binding protein
PCD4: programmed cell death 4
PVDF: polyvinylidene difluoride
RIF: radiation-induced intestinal fibrosis
TME: total mesorectal excision
